# Collaborative Localization in Wireless Sensor Networks *via* Pattern Recognition in Radio Irregularity Using Omnidirectional Antennas

**DOI:** 10.3390/s100100400

**Published:** 2010-01-06

**Authors:** Joe-Air Jiang, Cheng-Long Chuang, Tzu-Shiang Lin, Chia-Pang Chen, Chih-Hung Hung, Jiing-Yi Wang, Chang-Wang Liu, Tzu-Yun Lai

**Affiliations:** 1 Department of Bio-Industrial Mechatronics Engineering, National Taiwan University, Taipei 106, Taiwan; E-Mails: clchuang@ieee.org (C.C.); d98631001@ntu.edu.tw (T.L.); supercjb@pie.com.tw (C.C.); r97631035@ntu.edu.tw (C.H.); cloxy@pie.com.tw (J.W.); r97631027@ntu.edu.tw (C.L.); r97631040@ntu.edu.tw (T.L.); 2 Institute of Biomedical Engineering, National Taiwan University, Taipei 106, Taiwan

**Keywords:** localization, mobile applications, radiation pattern, received-signal strength, robust correlation, wireless sensor networks

## Abstract

In recent years, various received signal strength (RSS)-based localization estimation approaches for wireless sensor networks (WSNs) have been proposed. RSS-based localization is regarded as a low-cost solution for many location-aware applications in WSNs. In previous studies, the radiation patterns of all sensor nodes are assumed to be spherical, which is an oversimplification of the radio propagation model in practical applications. In this study, we present an RSS-based cooperative localization method that estimates unknown coordinates of sensor nodes in a network. Arrangement of two external low-cost omnidirectional dipole antennas is developed by using the distance-power gradient model. A modified robust regression is also proposed to determine the relative azimuth and distance between a sensor node and a fixed reference node. In addition, a cooperative localization scheme that incorporates estimations from multiple fixed reference nodes is presented to improve the accuracy of the localization. The proposed method is tested via computer-based analysis and field test. Experimental results demonstrate that the proposed low-cost method is a useful solution for localizing sensor nodes in unknown or changing environments.

## 1. Introduction

Wireless sensor networks (WSNs) [1–3] consist of a number of miniature low-power sensor nodes. The sensor nodes are mainly equipped with several micro-sensors, a microprocessor, and a radio chip with wireless communication capability. The functions of the sensor nodes that form WSNs are pretty diverse due to their wide and valuable applicability to various fields, and such functions also raise many topics of interest in the research field of wireless communication, e.g., energy-efficient routing [4] and sensing coverage problems [5]. Applications of WSNs have also stimulated great interest in developing wireless *ad hoc* sensor networks [6–7]. Unlike existing hardwired networks, the logical topology of a sensor network is not necessarily associated with its physical topology. In many cases, a sensor network is a data-centric system that measures the sensing events according to the attributes of the events. The data sensed by sensor networks are meaningless if we do not know the locations where the sensing events occur [8]. Thus, to provide a reliable localization scheme is an essential issue for the applications of WSNs when the location information of sensor nodes is required [9–12].

There are two easy ways to determine the location of each sensor node. The location information may be obtained while the network was deployed manually. The other approach is to equip each sensor node with a self-positioning device, e.g., a global positioning system (GPS) [13–16]. However, these methods are unrealistic to deploy a large-scale sensor network. Recently, many localization algorithms for WSNs have been proposed [17–34]. These algorithms can be categorized either as range-free or range-aware algorithms, based on whether they use the range information (i.e., distance) or not.

The range-aware approaches measure the distance between two sensor nodes based on physical measurements. Existing localization methods make use of four types of physical measurements: time of arrival (TOA) [17], time difference of arrival (TDOA) [18], angle of arrival (AOA) [19], and received signal strength (RSS) or energy [20–24]. These methods are mainly based on the measurements of acoustic ultrasounds or electromagnetic signals transmitted between sensor nodes. These approaches are found to have their own advantages and disadvantages [25]. Ultrasound-based TOA and TDOA estimations are not suitable for many practical applications due to signal-reverberating effects. A number of environmental factors, e.g., scattering, absorption, and reflection, may shorten the range of ultrasound propagation when an ultrasound wave encounters a particle that is small compared to its wavelength. These drawbacks make the ultrasound-based approaches unreliable. Radio-based TOA and TDOA estimations require high (up to nanosecond) synchronization accuracy for correct operation. On the other hand, measuring of AOA requires a set of carefully calibrated directional antennas, which significantly increases the cost and system complexity.

Because of the drawback of range-aware approaches, a number of range-free localization methods have been proposed, such as centroid [26], area-based point-in-triangulation [27], *ad hoc* positioning systems [28], convex position estimation [29], distributed localization estimation [30], Monte Carlo localization [31], and mobile [32,33] and static sensor network localization [34]. The error rates of range-free algorithms are high if the communication range of sensor nodes is not circular. In addition, the range-free algorithms require several sensor nodes working together to accomplish a localization task, so they suffer from power consumption issues. Among the approaches mentioned above, the radio propagation model is known as a simple function under *a priori* assumption. Such an assumption, however, is an oversimplification for many scenarios.

To address these challenges, we propose a localization framework for WSNs without adding expensive hardware (e.g., GPS, time synchronizer, and sensitive timer) to the sensor nodes. The basic principle of the proposed framework is to make use of the phenomenon of radio irregularity in WSNs using rotatable antennas. Rotatable antennas have been widely used in most of the AOA-based localization methods. However, the antennas used in those approaches are directional antennas. This is because directional antennas can concentrate energy on a particular narrow direction with a large gain. Therefore, most of recently proposed AOA-based localization methods were developed using directional antennas. The interference caused by surrounding noises can be reduced, and the localization accuracy was deemed an impracticable approach in the past. In this study, unlike other approaches, the major breakthrough is that we can achieve accurate localization of sensor nodes solely using omnidirectional antenna even if only one reference node exists. Besides, we can be benefit from the advantages of using omnidirectional antennas, e.g., low-cost (simplicity) and easy deployment (efficiency).

In this work, a robust correlation is incorporated in analyzing the relative positions between two sensor nodes using the received signal strength indication (RSSI) pattern. A cooperative localization scheme is also developed to improve the accuracy of the estimation as multiple reference nodes are available. The performance of the proposed framework has been evaluated by computer simulations and real world experiments under various experimental conditions.

The rest of this paper is organized as follows: Section 2 describes the definition of localization problems in WSNs, including network configuration, a pair of customized antenna modules, an azimuth dependent radio power model, and RSSI patterns. Section 3 presents the modified robust correlation to provide a better metric for matching RSSI patterns. Section 4 provides the collaborative localization scheme for precise localization. Experimental results yielded by computer simulation and field test are reported in Section 5. Finally, the discussion and conclusion are given in the last section.

## 2. Problem Formulation

### 2.1. Network Configuration

**Figure 1.**
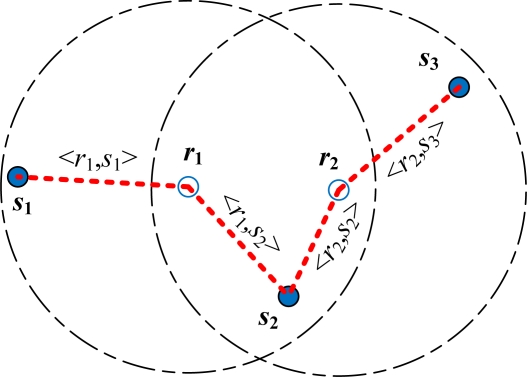
Architecture of a given sample network *G. G* = (*V*, *E*), where *V* = {*S*, *R*}, *S* = {*s*_1_, *s*_2_, *s*_3_}, *R* = {*r*_1_, *r*_2_}, and *E* = {<*r*_1_, *s*_1_>, <*r*_1_, *s*_2_>, <*r*_2_, *s*_2_>, <*r*_2_, *s*_3_>}.

Suppose a WSN is composed of sensor nodes and reference nodes that are deployed in a given sensing field. The objective of this study is to provide accurate location information of the sensor nodes in WSNs. The coordinates of the reference nodes are assumed known *a priori*. The location of the sensor node is estimated based on the measurements of nearby reference nodes. In this study, we focus on WSNs formed by a number of reference nodes that can estimate the locations of a given set of sensor nodes. Thus, we represent the network by the Euclidean graph *G* = (*V*, *E*), as depicted in Figure 1, with the following properties:

➢ *V* is a set of nodes in the network, and *V* = {*S*, *R*}; *S* is a set of sensor nodes equipped with RSSI sensors, and *S* = {*s*_1_, *s*_2_, …, *s_num_S_*}; *R* is a set of reference nodes equipped with servomotor-controlled external antennas, and *R* = {*r*_1_, *r*_2_, …, *r_num_R_*}. *num_S* is the number of sensor nodes; and *num_R* is the number of reference nodes.
➢ Sensor nodes *S* of the network do not know their location information.
➢ Physical positions of *R* are obtained by manual placement or external means. These nodes are the basis of the localization system.
➢ <*r_i_*, *s_j_*> ∈ *E*. It is sustainable if the distance between *r_i_* and *s_j_* is lesser than the communication range of *r_i_*.
➢ Given that a network *G* = (*V*, *E*) and *R* is with their physical position (*x_r_*, *y_r_*), for all *r* ∈ *R*, the goal of the localization system is to estimate the locations (*x_s_*, *y_s_*) of as many *s* ∈ *S*.

### 2.2. Configurations of External Antennas

In this study, all nodes *V* in the network *G* are equipped with an external omnidirectional dipole antenna. The omnidirectional antenna uniformly radiates power in the horizontal plane with a directional pattern shape in the vertical plane. These antennas are installed on *S* and *R* in different configuration that makes them be readily used in different operations.

**Figure 2.**
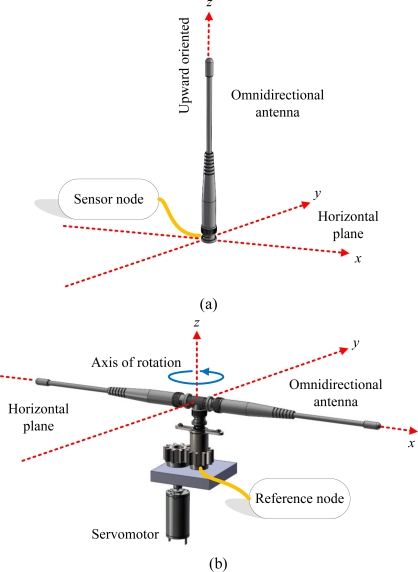
Schematic diagrams of the configurations used to couple external antennas and other peripheral circuits with (a) a sensor node and (b) a reference node.

*(1) Sensor nodes:* For each sensor node in *S*, an external antenna is coupled through an impedance matching circuit to the sensor module. The antenna is *z*-axis (upward) oriented in the vertical position to attain the best reception in any direction on the horizontal *xy*-plane. The schematic diagram of the sensor node mounted with external antenna is depicted in Figure 2(a). Note that no extra mechanism s required to control the antennas installed on sensor nodes.

*(2) Reference nodes:* With regard to the reference node in *R*, a low-power servomotor driven by a simple drive controller is installed. The schematic diagram of the reference node with external antenna is depicted in Figure 2(b). The servomotor is upward-oriented, which is perpendicular to the horizontal plane. Thus, the axis of rotation of the servomotor is perpendicular to the horizontal plane. By contrast, the antenna is oriented in the horizontal direction. The servomotor rotates against the *z*-axis at a constant angular speed of *v_c_* degrees per step counterclockwise. With this coupling mechanism, the radiation pattern of the reference node becomes directional on the horizontal *xy*-plane. Interestingly, this configuration is similar to a radar system, except that the radar uses electromagnetic waves to identify the distance and direction of a target, but the reference node in our localization system uses RSSI patterns. The cost of building this coupling mechanism is less than $60 US (including an omnidirectional antenna, stepper motor, motor control module, 8051 microcontroller, and battery), which makes the mechanism suitable for WSN applications.

### 2.3. Theoretical Justification of Antenna Configurations

Suppose that a sensor node *s* is located at an unknown location (*x_s_*, *y_s_*), and a reference node with an external antenna *r* is located at a known location (*x_r_*, *y_r_*). The goal of the localization problem is to estimate the unknown location of *s* by RSS measurements of a radio signal transmitted by *r*. The distance between *r* and *s* can be estimated based on the distance measurement by solving a system of nonlinear equations:

(1) $$d_{\langle r,s\rangle }=\sqrt{{\left(x_{r}-x_{s}\right)}^{2}+{\left(y_{r}-y_{s}\right)}^{2}}$$

where *d*_<*r*, *s*>_ is the measured distance between *r* and *s*. The reference node *r* broadcasts a beacon toward the sensor node *s* while the servomotor-controlled antenna of *r* rotates against the *z*-axis by *n* × *v_c_* degrees counterclockwise, where *n* is a gear ratio. The sensor node *s* measures the RSSI of the beacon from the reference node *r*, and transmits the measured RSSI back to *r*, immediately. The reference node *r* repeats above procedures on the condition that the sensor node *s* is still in the communication range of *r*.

The theoretical basis of RSSI measurements using the antenna configurations shown in Figure 2 is described as follows. From the *Friis equation*, the signal power of the beacon received by the sensor node *s* can be formulated by:

(2) $$\begin{array}{l} P_{s}\;\left(d_{\langle r,s\rangle },\theta_{s},\varphi_{s},{\vec{a}}_{s},\Gamma_{s},\theta_{r},\varphi_{r},{\vec{a}}_{r},\Gamma_{r}\right)\\ =P_{r}G_{s}\;\left(\theta_{s},\varphi_{s}\right)G_{r}\;\left(\theta_{r},\varphi_{r}\right){\left(\frac{\lambda }{\left(4\pi \right)d_{\langle r,s\rangle }}\right)}^{2}\left(1-{\left|\Gamma_{r}\right|}^{2}\right)\left(1-{\left|\Gamma_{s}\right|}^{2}\right){\left|{\vec{a}}_{r}\cdot {\vec{a}}_{s}^{*}\right|}^{2}\;e^{-\alpha d_{\langle r,s\rangle }} \end{array}$$

where *P_r_* is the signal power of the beacon transmitted by *r*, *P_s_* is the signal power of the beacon received by *s*, *λ* is the signal wavelength, and *α* is the attenuation coefficient of the mediums in the path of signal propagation. *G_r_* and *G_s_* are functions of angular directions that represent gains of the antenna of *r* and *s* in the direction (*θ_r_*, *φ_r_*) and (*θ_s_*, *φ_s_*), respectively. Γ*_r_* and Γ*_s_* are the reflection coefficients of the antennas of *r* and *s. a⃗_r_* and *a⃗_s_* are polarization vectors of the antennas of *r* and *s*, respectively. It clearly shows that *P_s_* is deeply influenced not only by *d*_<*r*, *s*>_, but also by the antenna orientations of *r* and *s*.

The spatial orientations of the antennas of *r* and *s* are in an orthogonal arrangement at all times regardless of the azimuths of the antenna of *r* against the *z*-axis. Based on the basic theory of radio wave propagation, the term ${\vec{a}}_{r}\cdot {\vec{a}}_{s}^{*}$ in Equation (2) is zero due to that the polarization vector of the antennas of *r* and *s* are mismatch. Theoretically, the term ${\vec{a}}_{r}\cdot {\vec{a}}_{s}^{*}$ deflates the value of *P_s_* to zero; therefore, no beacon can be received by *s*. However, in real world scenario, two devices are still able to exchange information via electromagnetic waves even if their antennas are in orthogonal arrangement. Obviously, the polarization of the electromagnetic (EM) wave that carries the beacon somehow can be altered by environmental factors (e.g., particles or interfaces) existing in real world experiments. Therefore, before we introduce the methodology part of this study, we need to build a theoretical foundation to justify that the proposed antenna configuration is applicable.

**Figure 3.**
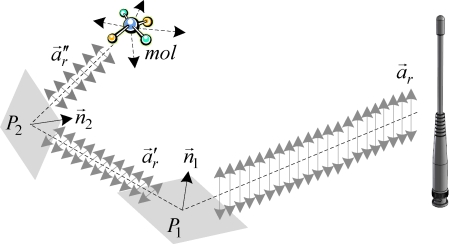
Example of alteration of polarization state of an EM wave.

Many media and interfaces can function affect the polarization of the EM wave. According to the *Brewster’s law*, when the EM wave reflects at an incidence angle from a non-metallic (dielectric) interface, it results in a polarized EM wave. All reflected radio signal must be *s*-polarized with an electrical field parallel to the interface [35]. Thus, if a polarized EM wave reflects from a dielectric interface, the component of the electrical field perpendicular to the reflection interface is selectively refracted. This achieves a rotation of the polarization vector of the reflected EM wave. Adding more reflection interfaces in the propagation path of the EM wave, the polarization angle of the EM wave can be altered to all possible angles, which follows the *Law of Malus* [36]. As an example of radiation propagation shown in Figure 3, the antenna of the reference node broadcasts a beacon carried by an EM wave with the polarization vector *a⃗_r_*. The polarization vector *a⃗_r_* is altered to *a⃗*′*_r_* after the EM wave reflects from an plane *P*_1_ that has the normal vector *n⃗*_1_. Again, *a⃗*′*_r_* is altered to *a⃗*″*_r_* after the EM wave reflects from an plane *P*_2_ that has the normal vector *n⃗*_2_. The EM wave is scattered to all directions if it encounters small molecules of the air, known as the *Rayleigh scattering* [37]. Thus, the EM wave that has altered polarization vector *a⃗*″*_r_* can propagate to all possible directions. Thereby, the beacon transmitted by the reference node can be received by the antenna of the sensor node regardless of whether the polarization vectors {*a⃗_r_*, *a⃗_s_*} are matched or not.

According to the descriptions given above, we suppose that any existing interface in the natural environment functions as an action on the polarization vector (*a⃗_r_*) of the EM wave. Assuming that there are *N_p_* interfaces (*P_i_*’s) given by:

(3) $$P_{i}:a_{i}x+b_{i}y+c_{i}z=d$$

where *i* = 1, …, *N_p_*, and *P_i_* can be represented as the plane for manipulating the polarization vector of an incidence EM wave. Suppose that a beacon signal encounters an interface *P_i_* with the incidence vector *v⃗_inc_*. The reflection vector of *P_i_* can be calculated by:

(4) $${\vec{v}}_{\mathrm{ref}}={\vec{v}}_{\mathrm{inc}}-2\left({\vec{v}}_{\mathrm{inc}}\cdot {\vec{n}}_{i}\right){\vec{n}}_{i}$$

where *n⃗_i_* is the unit normal vector of *P_i_* that can be formulated by:

(5) $${\vec{n}}_{i}=\frac{\left(a_{i},b_{i},c_{i}\right)}{\sqrt{a_{i}^{2}+b_{i}^{2}+c_{i}^{2}}}$$

The EM wave is then re-polarized in a new direction:

(6) $${{\vec{a}}^{\prime }}_{r}={\vec{v}}_{\mathrm{ref}}\times {\vec{n}}_{i}$$

According to the *Law of Malus*, the amplitude of the reflected EM wave is:

(7) $$E_{\mathrm{ref}}=E_{\mathrm{inc}}\times \text{cos}\;\theta_{{\vec{a}}_{r},{{\vec{a}}^{\prime }}_{r}}$$

where *E_ref_* and *E_inc_* are the amplitude of the reflected EM wave and the incidence EM wave, respectively. *θ*_*a⃗*_*r*_, *a⃗*′_*r*__ is the angle between *a⃗_r_* and *a⃗*′*_r_*, thereby cos *θ*_*a⃗*_*r*_, *a⃗*′_*r*__ can be obtained as:

(8) $$\text{cos}\;\theta_{{\vec{a}}_{r},{{\vec{a}}^{\prime }}_{r}}=\frac{{\vec{a}}_{r}\cdot {{\vec{a}}^{\prime }}_{r}}{\left\|{\vec{a}}_{r}\right\|\left\|{{\vec{a}}^{\prime }}_{r}\right\|}$$

With the aforementioned formulation, we assume that an EM wave with the electric field *E*_0_ is emitted from an antenna of a reference node. The antenna is horizontal oriented with a polarization vector parallel to the horizontal plane. All interfaces are randomly presented in the pseudo-space with random orientations. The EM wave uniformly propagates through the air and encounters a random number of interfaces. Assume here that there will be between 1 to 100 random incidence vectors. By performing a computer simulation, the amplitude of the electric field of the EM wave that its polarization vector (denoted by ${\vec{a}}_{r}^{\left(n\right)}$) is perpendicular to the horizontal plane is *E_h_* = 0.0076 *E*_0_. Since the antenna of the sensor node is vertically oriented, it can receive the multi-reflected EM wave with the polarization vector ${\vec{a}}_{r}^{\left(n\right)}$. As the antenna of the sensor node is fixed at upward orientation, the electric field that can be detected by the antenna is roughly 1.3439 × 10^−5^ *E*_0_.

With the derivation given above, we assume that the orientations of incident surfaces existing in the natural environment are randomly oriented, the term ${\left|{\vec{a}}_{r}\cdot {\vec{a}}_{s}^{*}\right|}^{2}$ can be reformulated as an approximation form:

(9) $${\left|{\vec{a}}_{r}\cdot {\vec{a}}_{s}^{*}\right|}^{2}≅{\left|\sum_{n}{\vec{a}}_{r}^{\left(n\right)}\cdot {\vec{a}}_{s}^{*}/n\right|}^{2}$$

where ${\vec{a}}_{r}^{\left(n\right)}$ and *a⃗_s_* are the polarization vectors of the multi-reflected EM wave and the antenna of the sensor node, respectively. If there is a strong multipath effect, *a⃗_r_* can be reoriented to ${\vec{a}}_{r}^{\left(n\right)}$ that is partially detectable by the antenna of the sensor node with the polarization vector *a⃗_s_*. Thus, the sensor node *s* is still able to receive the beacon transmitted from the reference node *r* in the natural environment, no matter whether the polarization vectors of the antennas of *s* and *r* are orthogonal or not. The term ${\left|{\vec{a}}_{r}\cdot {\vec{a}}_{s}^{*}\right|}^{2}$ can be reduced to a constant *c_a_*.

Regarding the reflection coefficients Γ*_r_* and Γ*_s_*, they describe the ratio of reflection while the EM wave reaches the antenna of *s*. Since Γ*_r_* and Γ*_s_* are angle invariant scalars, the term (1 – |Γ*_r_*|^2^) · (1 – |Γ*_s_*|^2^) in Equation (2) is reduced to a constant *c*_Γ_. In addition, the mediums in the path of signal propagation are mainly air. The attenuation coefficient *α* of clear air is 0.0003 m^−1^ according to [38]. Thus, the *Friis equation* can be approximated by setting *α* at near zero, and the term *e*^−*αd*_〈*r,s*〉_^ can be completely reduced to a constant *c_α_* ≅ 1.

The signal wavelength *λ* is a fixed value. In order to simplify the problem, we assume that all antennas are positioned at the same height. The orientation of the omnidirectional antenna of the sensor node *s* is upward oriented, this fact leads *G_s_*(*θ_s_*, *φ_s_*) to a fixed value. Thus, the effects of *θ_s_* and *φ_s_* can be further omitted. The antenna of the reference node *r* is an omnidirectional one. *φ_r_* can be omitted since the gain of the antenna is a function that simply depends on *d*_<*r*, *s*>_ and *θ_r_*. With the aforementioned facts, the *Friis equation* in Equation (2) can be expressed by a more compact form as:

(10) $$P_{s}\;\left(d_{\langle r,s\rangle },\theta_{r}\right)=P_{r}G_{s}G_{r}\;\left(\theta_{r}\right)\;{\left(\frac{\lambda }{\left(4\pi \right)d_{\langle r,s\rangle }}\right)}^{2}c_{\Gamma }c_{a}c_{\alpha }$$

Therefore, the variables that are able to manipulate *P_s_* are *d*_<*r*, *s*>_ and *θ_r_*. The RSSI determined by a sensor node *s* is a measurement of power presented in a beacon broadcasted by a reference node *r*. It measures the signal power in dB unit. According to the simplified *Friis equation* in Equation (10), we can approximate the theoretical model of RSSI by transforming the simplified *Friis equation* into log-space:

(11) $$\text{log}\;P_{s}\;\left(d_{\langle r,s\rangle },\theta_{r}\right)=\text{log}\;P_{r}+\text{log}\;G_{r}\;\left(\theta_{r}\right)-2\;\text{log}\;d_{\langle r,s\rangle }+\text{log}\;c$$

where *c* = *G_s_*·*c*_Γ_·*c_a_*·*c_α_*·(*λ*/(4*π*))^2^, and *c* represents the shadow fading effects produced by the multipath environment. By comparing log*P_s_*(*d*_<*r*, *s*>_, *θ_r_*) with the classic path loss model of narrowband radio propagation, the proposed antenna configurations can reflect the changes in *θ_r_*. For a given network, log*P_s_*(*d*_<*r*, *s*>_, *θ_r_*) can be calculated or measured during the period of system calibration, and log*P_r_* and log*G_s_*(*θ_r_*) can be determined in real-time at the reference node. If the transmitted power *P_r_* is fixed, *d*_<*r*, *s*>_ and *θ_r_* can be used to determine the position and azimuth of *s* relative to *r*.

### 2.4. RSSI Pattern

While the antenna of the reference node *r* rotates against the *z*-axis, the measured RSSI changes along with *θ_r_*. As previously mentioned, the reference node *r* broadcasts a beacon while the antenna of *r* rotates by *n_g_* × *v_c_* degrees counterclockwise, where *n_g_* represents the gear ratio. A complete RSSI pattern for *r* and *s* is formed by transmitting the beacon for 2*π*/(*n* × *v_c_*) times over *δ*, where *δ* is the azimuth of *s* relative to *r*. The RSSI pattern can be formulated by:

(12) $$\Omega_{\langle r,s\rangle }\;\left(\delta \right)=\Lambda_{r}\;\left(\delta \right)+\varepsilon ,\;\delta \in \left\{n_{g}v_{c},\;2n_{g}v_{c},\;...,\;2\pi \right\}$$

where Ω_<_*_r_*_,_ *_s_*_>_(*δ*) is the RSSI pattern, Λ*_r_*(*δ*) = log*G_r_*(*δ*), and *ε* = log*P_r_* – 2log*d*_<*r*, *s*>_ + log*c*.

**Figure 4.**
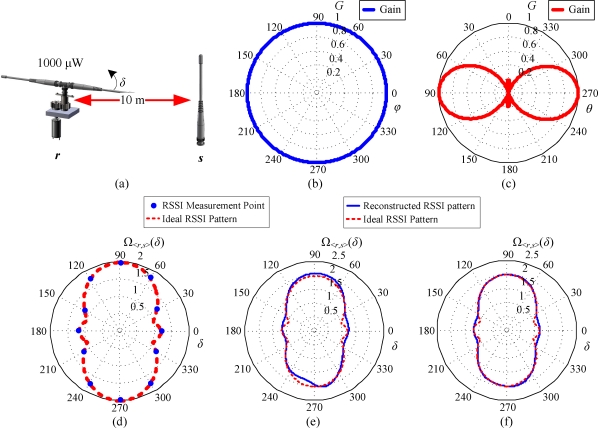
An example of RSSI measurement. (a) A pseudo scenario that consists of a sensor node *s* and a reference node *r*, where the sensor node is located at the eastern side of the reference node and the angle of rotation of the antenna of the reference node is denoted by *δ*; (b) The H-plane EM wave pattern of the omnidirectional antenna utilized in this study; (c) The E-plane EM wave pattern of the omnidirectional antenna utilized in this study; (d) An ideal RSSI pattern and RSSI measurement points that are derived from Equation (2); (e) A reconstructed RSSI pattern after the antenna of the reference node completes the first cycle of rotation; (f) A stabilized RSSI pattern that is estimated by repeated RSSI measurements.

For an example given in Figure 4(a), we suppose that a sensor node *s* and a reference node *r* are separated 10 meters, and *s* is located at the eastern side relative to *r*. The servomotor-controlled antenna of *r* transmits a beacon at the power level of 0 dBm. In this case, let *P_r_* = 1,000 μW, *d*_<*r*, *s*>_ = 10 m, and *c* ∼ *N*(1, 0.01), where *N* denotes normal distribution, and *ε* = 1 + log*c* at all time. The stepping angle of the servomotor is assumed to be 1° per step (*v_c_* = 1 degree/step). The reference node *r* transmits a beacon toward the sensor node *s* while the antenna of *r* rotates by 30 degrees (*n_g_* × *v_c_* = 30). After the antenna of *r* completes a full circle of rotation, 12 RSSIs are measured. The EM wave pattern of the antenna of *s* and *r* in the H-plane is assumed to be an ideal circular pattern as shown in Figure 4(b). The EM wave pattern of the antennas in the E-plane is assumed to be a pattern of five-element array, which is depicted in Figure 4(c). Since the antenna of *s* is upward oriented, the EM wave pattern of *s* in the horizontal plane is identical to that in the H-plane. On the other hand, as the antenna of *r* is oriented toward the horizontal direction, its EM wave pattern Λ*_r_*(*δ*) in the horizontal plane is the antenna pattern in E-plane. A set of ideal RSSI measurement points and an ideal RSSI pattern acquired by Equation (12) are illustrated in Figure 4(d). With the consideration of noise caused by the multipath effect, an RSSI pattern Ω_<_*_r_*_,_ *_s_*_>_(*δ*) can be reconstructed after the antenna of *r* completes one circle of rotation. As shown in Figure 4(e), the reconstructed pattern is slightly different from the ideal pattern due to the insufficient measurement points. The reconstructed RSSI pattern also suffers from multipath distortion. In Figure 4(f), a more precise RSSI pattern Ω_<_*_r_*_,_ *_s_*_>_(*δ*) related to the ideal one can be acquired by averaging the RSSIs obtained from repeat measurements. The reconstructed RSSI pattern is more precise because the repeat measurements improve the signal-to-noise ratio of the pattern. This pattern clearly shows that the sensor node *s* is located at the western or eastern side relative to the reference node *r*. Now, the problem of localization estimation is formulated into a nonlinear equation with unknown parameters *d*_<*r*, *s*>_ and *δ*. In the next section, a robust solution specifically designed for this problem is presented.

## 3. Localization Using Robust Correlation Estimator

**Figure 5.**
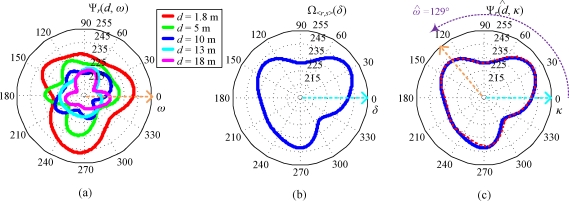
Examples of RSSI patterns. (a) Reference standard RSSI patterns Ψ*_r_*(*d*, *ω*) of a given reference node and five patterns measured when the sensor node and the reference node are distanced by 1.8 m, 5 m, 10 m, 13 m, and 18 m; (b) An RSSI pattern Ω_<_*_r_*_,_ *_s_*_>_(*δ*) between the aforementioned reference node and a sensor node with unknown coordinates; (c) By matching Ω_<_*_r_*_,_ *_s_*_>_(*δ*) with Ψ*_r_*(*d*, *ω*), the distance and angular direction of the sensor node relative to the reference node estimated at 1.8 m and 129° counterclockwise, respectively.

Assume that the RSSI patterns of any given paired nodes *s* and *r* at all possible distances *d* are known a priori. These patterns are served as reference standard RSSI patterns Ψ*_r_*(*d*, *ω*), where *ω* is the azimuths of antenna of *r*. A sample pattern Ψ*_r_*(*d*, *ω*) measured by real-world experiments under the condition that *s* is located at the northern side relative to *r* is illustrated in Figure 5(a). We can see that these patterns are asymmetric due to the effect of radio irregularity, which is quite different from the ideal examples given in the previous section. However, we can benefit from the asymmetric pattern in Ψ*_r_*(*d*, *ω*), because it provides us more information of the pattern at different angle *ω*. For instance, if Ψ*_r_*(*d*, *ω*) is symmetric as the ideal example in Figure 4(d), we can precisely determine the distance between *r* and *s*, but the orientation angle of *s* relative to *r* is still uncertain. This problem is eliminated if Ψ*_r_*(*d*, *ω*) is constructed by asymmetric patterns. By matching Ω_<_*_r_*_,_ *_s_*_>_(*δ*) with Ψ*_r_*(*d*, *ω*), the distance and direction of a given sensor node *s* relative to a reference node *r* can be estimated.

Given an unknown distance between *r* and *s*, an RSSI pattern Ω_<_*_r_*_,_ *_s_*_>_(*δ*) can be obtained. A sample of RSSI pattern Ω_<_*_r_*_,_ *_s_*_>_(*δ*) measured between a reference node and a sensor node with unknown coordinate is depicted in Figure 5(b). Now, the problem is that for a known Ψ*_r_*(*d*, *ω*) we need to estimate two variables, *d̂* and *ω̂* to minimize the difference between Ψ*_r_*(*d̂*, *ω*) and Ω_<_*_r_*_,_ *_s_*_>_(*δ* – *ω̂*), where *d̂* can be interpreted as the potential distance between *r* and *s*, and *ω̂* can be interpreted as a potential orientation angle of *s* relative to *r*, counterclockwise.

Many well-known metrics (e.g., Euclidian distance, Pearson correlation) have been proposed for pattern matching. These metrics are proven effective in solving linear problems, but they do not work well in nonlinear cases, nor do they in handling data with outliers. While the distance between *s* and *r* is fixed, Ψ*_r_*(*d*, *ω*) and Ω_<_*_r_*_,_ *_s_*_>_(*δ*) are nonlinear functions of azimuths *ω* and *δ* with noises at an uncertain level (e.g., the height of a sensor node). Thus, matching RSSI patterns is a highly nonlinear problem so that linear metrics are inapplicable to this case. In this study, we develop a metric, named ‘robust correlation estimator’, to indicate the relation between two nonlinear functions, Ψ*_r_*(*d*, *ω*) and Ω_<_*_r_*_,_ *_s_*_>_(*δ*).

First, we need to recognize that the RSSI patterns Ψ*_r_*(*d*, *ω*) and Ω_<_*_r_*_,_*_s_*_>_(*δ*) are functions of the angular direction *ω* and *δ*. It means that they are measured depending on the rotation angle of the antenna of *r*. Thus, when we compare two RSSI patterns, it is necessary to consider the information merged in *ω* and *δ*. Under this concept, we take first-order partial derivatives of Ψ*_r_*(*d*, *ω*) and Ω_<_*_r_*_,_ *_s_*_>_(*δ*) with respect to *ω* and *δ*, respectively, which can be derived as:

(13) $$\nabla \psi_{r}\;\left(d,\omega \right)=\frac{\partial \psi_{r}\;\left(d,\omega \right)}{\partial \omega }=\psi_{r}\;\left(d,\omega +1\right)-\psi_{r}\;\left(d,\omega \right)$$

(14) $$\nabla \Omega_{\langle r,s\rangle }\;\left(\delta \right)=\frac{\partial \Omega_{\langle r,s\rangle }\;\left(\delta \right)}{\partial \delta }=\Omega_{\langle r,s\rangle }\;\left(\delta +1\right)-\Omega_{\langle r,s\rangle }\;\left(\delta \right)$$

where ▿Ψ*_r_*(*d*, *ω*) and ▿Ω_<_*_r_*_,_ *_s_*_>_(*δ*) represents the first-order derivative of Ψ*_r_*(*d*, *ω*) and Ω_<_*_r_*_,_ *_s_*_>_(*δ*), respectively. The purpose of this step is to preserve the relationship between two RSSIs measured at neighboring angles. In addition, the features of RSSIs measured at adjoining azimuths can be observed during the matching process.

Furthermore, we use a linear regression model to fit ▿Ψ*_r_*(*d*, *κ*) and ▿Ω_<_*_r_*_,_ *_s_*_>_(*κ*) by:

(15) $$\nabla \psi_{r}\;\left(\hat{d},\kappa \right)=\beta_{0}+\beta_{1}\;\left(\hat{d},\hat{\omega }\right)\nabla \Omega_{\langle r,s\rangle }\;\left(\kappa +\hat{\omega }\right)+\varepsilon \left(\beta_{0},\beta_{1},\kappa \right)$$

where *d̂* is the potential distance between *r* and *s*, *κ* is a dummy variable ranged from 0 to 2π, *ω̂* is the azimuth of *s* relative to *r*, *ε*(*β*_0_, *β*_1_, *κ*) is the disturbance term, and *β*_0_ and *β*_1_ are the intercept and slope of the regression line, respectively. Since the first-order derivative step neutralizes the baseline shift effect, the intercept *β*_0_ can be removed from Equation (15). In this study, the disturbance term *ε*(*β*_1_, *κ*) is reformulated by Cauchy-Lorentz distribution [39] to reduce the influences of outliers, which is given by:

(16) $$\varepsilon^{*}\left(\hat{d},\hat{\omega },\beta_{1},\kappa \right)=\frac{1}{1+{\left(\nabla \psi_{r}\;\left(\hat{d},\kappa \right)-\beta_{1}\;\left(\hat{d},\hat{\omega }\right)\nabla \Omega_{\langle r,s\rangle }\;\left(\kappa +\hat{\omega }\right)\right)}^{2}}$$

Since we reformulated the disturbance term *ε* into a reweighted one ε^*^ based on the Cauchy-Lorentz distribution function, the data points that fit well to the model in Equation (15) produce larger ε^*^, and the data points that do not fit well to the model give lower ε^*^. Consequently, the optimal slope *β̂*_1_(*d̂*, *ω̂*) of the regression line fitted to the data can be obtained by maximizing the sum of ε^*^, iteratively. The goal of the robust correlation estimator is to estimate *β*_1_ by maximizing the sum of ε^*^(*d̂*, *ω̂*, *β*_1_, *κ*) for *κ* = 0, …, 2*π*, and *β*_1_ can be formulated as:

(17) $${\hat{\beta }}_{1}\;\left(\hat{d},\hat{\omega }\right)=\underset{\beta_{1}}{\text{arg}\;\text{max}}\sum_{\kappa =0}^{2\pi }{\left(\varepsilon^{*}\;\left(\hat{d},\hat{\omega },\beta_{1},\kappa \right)\right)}^{2}$$

Since the value of *β̂*_1_(*d̂*, *ω̂*) is in an interval ranging from –∞ to ∞, we use the variances of ▿Ψ*_r_*(*d*, *ω*) and ▿Ω_<_*_r_*_,_ *_s_*_>_(*κ*) to normalize the value into an interval ranging from −1 to 1 that allows for better interpretation and analysis. To transform *β̂*_1_(*d̂*, *ω̂*) into an interval ranging from −1 to 1, a coefficient of the robust correlation *τ*(*d̂*, *ω̂*) can be obtained by:

(18) $$\tau \left(\hat{d},\hat{\omega }\right)=\{\begin{array}{cc} \frac{{\hat{\beta }}_{1}\;\left(\hat{d},\hat{\omega }\right)}{\sigma^{*}\;\left(\nabla \psi_{r}\;\left(\hat{d},\kappa \right),\nabla \Omega_{\langle r,s\rangle }\;\left(\kappa \right)\right)} & \text{if}\;\left|{\hat{\beta }}_{1}\;\left(\hat{d},\hat{\omega }\right)\right|\leq 1\\ \frac{1/{\hat{\beta }}_{1}\;\left(\hat{d},\hat{\omega }\right)}{\sigma^{*}\left(\nabla \psi_{r}\;\left(\hat{d},\kappa \right),\nabla \Omega_{\langle r,s\rangle }\;\left(\kappa \right)\right)} & \text{otherwise} \end{array}$$

where σ^*^(▿Ψ*_r_*(*d*, *κ*), ▿Ω_<_*_r_*_,_ *_s_*_>_(*κ*)) is the scaling factor for the transformation, which is defined as:

(19) $$\sigma^{*}\left(\nabla \psi_{r}\;\left(\hat{d},\kappa \right),\nabla \Omega_{\langle r,s\rangle }\;\left(\kappa \right)\right)=\text{max}\left(\frac{\sigma \left(\nabla \psi_{r}\;\left(\hat{d},\kappa \right)\right)}{\sigma \left(\nabla \Omega_{\langle r,s\rangle }\;\left(\kappa \right)\right)},\;\frac{\sigma \left(\nabla \Omega_{\langle r,s\rangle }\;\left(\kappa \right)\right)}{\sigma \left(\nabla \psi_{r}\;\left(\hat{d},\kappa \right)\right)}\right)$$

where *σ*(▿Ψ*_r_*(*d*, *κ*)) and *σ*(▿Ω_<_*_r_*_,_ *_s_*_>_(*κ*)) are variances of ▿Ψ*_r_*(*d*, *κ*) and ▿Ω_<_*_r_*_,_ *_s_*_>_(*κ*), respectively. The amplitude of *τ*(*d̂*, *ω̂*) measures the strength of similarity between ψ*_r_*(*d̂, κ*) and Ω_<_*_r_*_,_ *_s_*_>_(*κ* + *ω̂*). For instance, *r* and *s* are likely distanced *d̂* meters apart when *τ*(*d̂*, *ω̂*) = 1, and the angular direction of *s* relative to *r* is *ω̂*, counterclockwise. In addition, *τ*(*d̂*, *ω̂*) *=* 0 means that there is no relation between these two-paired RSSI patterns. As shown in Figure 5(c), by matching Ω_<_*_r_*_,_ *_s_*_>_(*δ*) with Ψ*_r_*(*d*, *ω*), we can obtain a large value of *τ*(*d̂*, *ω̂*) which is equal to 0.97 if *d̂*= 1.8 and *ω̂*= 129° are given.

The localization problem now can be formulated by a maximum function as:

(20) $$\left(d_{\langle r,s\rangle },\omega_{\langle r,s\rangle }\right)=\underset{\hat{d},\hat{\omega }}{\text{arg}\;\text{max}}\;\tau (\hat{d},\hat{\omega })$$

where *d*_<_*_r_*_,_ *_s_*_>_ is the predicted distance between *r* and *s*, and *ω*_<_*_r_*_,_ *_s_*_>_ is the predicted angular direction of *s* relative to *r*, counterclockwise. Thus, if the coordinate of *r* is (*x_r_*, *y_r_*), the coordinate of *s* can be predicted by (*x_s_*, *y_s_*), and (*x_s_*, *y_s_*) = (*x_r_* + *d*_<_*_r_*_,_ *_s_*_>_cos(*ω*_<_*_r_*_,_ *_s_*_>_), *y_r_* + *d*_<_*_r_*_,_ *_s_*_>_sin(*ω*_<_*_r_*_,_ *_s_*_>_)). The robust correlation estimator proposed in this section can be used to analyze the similarity or dissimilarity of RSSI patterns in multidimensional space. It allows the network to locate the position of a sensor node through a fixed reference node.

## 4. Collaborative Localization Scheme Using Multiple Reference Nodes

The localization method proposed in Section 3 directly converts the problem into the framework of collaborative localization when multiple reference nodes are considered. Based on the result in Equation (20), when multiple reference nodes cover the same sensor node, the geometric positions estimated by multiple measurements can be used to improve the accuracy of the localization. In this section, a collaborative localization scheme is presented to perform this task.

Suppose that there is a sensor node *s* covered by *n* reference nodes *r*_1_, *r*_2_, …, and *r_n_*. Each reference node broadcasts a series of beacons toward the sensor node for measuring RSSI patterns. By matching the RSSI patterns with the reference standard patterns of reference nodes using the method presented in the last section, we can obtain the robust correlation coefficients by:

(21) $$\forall i=1,...,n,\psi_{r_{i}}\;\left(d,\omega \right)\overset{\text{Matching}}{↔}\Omega_{\langle r_{i},s\rangle }\;\left(\delta \right)\to \tau_{\langle r_{i},s\rangle }\;\left({\hat{d}}_{i},{\hat{\omega }}_{i}\right)$$

where *d̂_i_* and *ω̂_i_* are the potential distance and angular direction of *s* relative to *r_i_*. All robust correlations are merged together into one overall solution space in accordance with the coordinates of the reference nodes. For all robust correlations *τ*_〈*r*_*i*_, *s*〉_(*d̂_i_, ω̂_i_*), *i* = 1, 2, …, *n*. We convert them into a two-dimensional Cartesian coordinate system by:

(22) $$\begin{array}{l} \forall \;\;{\hat{d}}_{i}\text{ and }{\hat{\omega }}_{i},\\ {ϒ}_{\langle r_{i},s\rangle }\;\left(x_{r_{i}}+{\hat{d}}_{i}\;\text{cos}\;{\hat{\omega }}_{i},\;y_{r_{i}}+{\hat{d}}_{i}\;\text{sin}\;{\hat{\omega }}_{i}\right)=\tau_{\langle r_{i},s\rangle }\;\left({\hat{d}}_{i},{\hat{\omega }}_{i}\right) \end{array}$$

We set the initial values in an overall solution space ℑ(*x, y*) at one. The merging process of all robust correlations can be formulated by:

(23) ∀ (x,y) in ϒ〈ri,s〉 (x,y), where i=1, 2, ..., n,{ℑ(xri+x,yri+y)=ℑ(xri+x,yri+y)×ϒ〈ri,s〉 (x,y)      if x2+y2≤ℜℑ(xri+x,yri+y)=0                                                   otherwise

where (*x*_*r*_*i*__, *y*_*r*_*i*__) is the coordinate of the reference node *r_i_*, and ℜ is the reliable localization capability of the reference nodes. The range of ℜ can be determined by the range of *d* in the reference standard patterns Ψ*_r_*(*d*, *ω*) of the reference node *r*.

After the overall solution space is obtained, we can determine the highest possible position of the sensor node *s* using the squared-centroid of a set of projected points in ℑ(*x*, *y*) as:

(24) $$\begin{array}{l} \forall \;\left(x,y\right)\;\text{in}\;ℑ\left(x,y\right),\\ {\hat{x}}_{s}=\frac{\sum_{x}{\left(\underset{y}{\text{max}}\;ℑ\left(x,y\right)\right)}^{2}\cdot x}{\sum_{x}{\left(\underset{y}{\text{max}}\;ℑ\left(x,y\right)\right)}^{2}},\;{\hat{y}}_{s}=\frac{\sum_{y}{\left(\underset{x}{\text{max}}\;ℑ\left(x,y\right)\right)}^{2}\cdot y}{\sum_{y}{\left(\underset{x}{\text{max}}\;ℑ\left(x,y\right)\right)}^{2}} \end{array}$$

where (*x̂_s_*, *ŷ_s_*) is the estimated coordinate of the sensor node *s*. Since the squared-centroid method is linear computational complexity (*x* + *y*), it is more preferred than a traditional centroid method with an order of (*x* × *y*) time complexity. With more reference nodes involved in the localization process, we can further improve the accuracy of coordinate estimation presented above.

## 5. Experimental Results

In this section, we evaluate the performance of the proposed RSS-based cooperative localization method using two examinations, computer simulations in MATLAB and real-world field experiments. In the computer simulation case, we compare the performance of the proposed method with the results published in [22]. For comparison, simulation parameters are set at the values identical to [22] as summarized in Table 1. To apply these parameters, an ordinary log-distance path loss model, which can be formulated as:

(25) $$P_{L}=P_{r}-P_{s}=P_{0}+10\gamma \;{\text{log}}_{10}\;\frac{d_{\langle r,s\rangle }}{d_{0}}+\varepsilon_{g}$$

is used to evaluate the methods in [22]. In Equation (25)*P_L_* is the total path loss, *P_r_* is the signal power of the beacon transmitted by *r*, *P_s_* is the signal power of the beacon received by *s*, *P*_0_ is the path loss at the reference distance when *d*_0_ = 1, *d*_<*r*, *s*>_ is the measured distance between *r* and *s*, and *ε_g_* is Gaussian random noise with zero mean, reflecting fading due to multipath propagation or shadowing from obstacles affecting the wave propagation.

However, given the antenna configuration proposed in this study, the ordinary log-distance path loss model is insufficient to model the behavior of wave propagation between reference nodes and sensor nodes. Therefore, we modify the path loss model in Equation (25) as below:

(26) $$P_{L}=P_{r}-P_{s}=P_{0}+10\gamma G_{s}G_{r}\;\left(\theta_{r}\right)\;{\text{log}}_{10}\;\frac{d_{\langle r,s\rangle }}{d_{0}}+\left(\varepsilon_{g}+\sum_{r_{i}\in R}\varepsilon_{\langle r_{i},s\rangle }\right)$$

where the interference of reference nodes *ε*_〈*r*_*i*_, *s*〉_ is formulated by Gaussian noise controlled by envelop amplitudes *G_r_*(*θ*_*r*_*i*__), which is given as below:

(27) $$\varepsilon_{\langle r_{i},s\rangle }=G_{r}\;\left(\theta_{r_{i}}\right)\;\varepsilon_{g}$$

Therefore, the interference model in Equation (27) takes the impact of the antenna angles (*θ*_*r*_*i*__) of distinct reference nodes into consideration during the simulation study, which provides a more accurate channel-model than using Gaussian model as in [22]. Thus, the proposed method is examined under a stringent path loss model in Equations (26) and (27), while the other methods for comparison use the simpler one in Equation (25).

In real world scenarios, the results yielded by the proposed algorithm are merely conducted from field measurements of RSSI patterns. Thereby, the actual parameter values in real-world scenarios are not required for the localization process using the proposed algorithm.

### 5.1. Performance Evaluations using Computer Simulations

**Figure 6.**
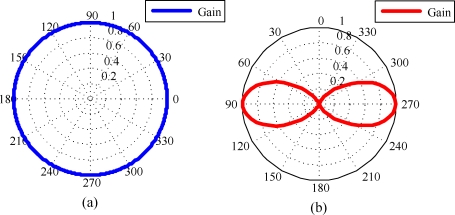
The radiation pattern of the antenna of *s* and *r* (a) The H-plane radiation pattern of the omnidirectional antenna utilized in the simulation. (b) The E-plane radiation pattern of the omnidirectional antenna utilized in the simulation.

**Figure 7.**
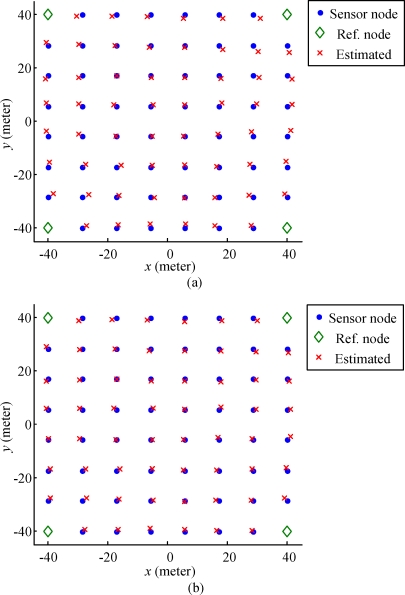
Actual locations of the deployed sensor nodes and the reference nodes, as compared to the estimated locations of the sensor nodes with (a) one rotation cycle and (b) two cycles of the antenna on the reference nodes.

The radiation pattern of the antenna of *s* and *r* in the H-plane is assumed to be an ideal circular pattern as shown in Figure 6(a). The radiation pattern of the antennas in the E-plane is assumed to be a pattern of three-element array, which is depicted in Figure 6(b). In order to avoid losing the generality of the simulation, we utilize a uniform-grid deployment structure of nodes in a square sensing field, as shown in Figure 7(a). There are four reference nodes deployed at the corner points, and all sensor nodes are deployed at other grid points. Such a deployment structure also allows us to visualize the simulation results of individual sensor nodes. The reference standard patterns of the four reference nodes are generated using the ideal radio model defined in Equation (12). In all of the RSSI patterns, the reference standard patterns Ψ*_r_*(*d*, *ω*) can be measured in the conditions that *d* = 1∼100 m (with interval of 0.1 m) and *ω* = 1°∼360° (with interval of 1°). In the localization process, we also use the definition in Equation (12) to simulate the measured RSSI pattern Ω_<_*_r_*_,_ *_s_*_>_(*δ*) with noise interference *ε_g_* ≈ *N*(0, 6), which is identical to [22].

For each sensor node, the reference node performs a complete measurement of the RSSI pattern by rotating the antenna, counterclockwise. The results yielded by the proposed algorithm are shown in Figure 7(a). In 100 repeated test cases (with non-synchronized antenna rotation speeds and rotation angles), the averaged bias is 1.89 m, and the standard deviation of the bias is 1.31 m. The sensor nodes with larger estimation biases are distributed around the four corners, in which the maximum estimation error is 4.78 m. The nodes with lesser estimation errors are mostly located at the center of the sensing field covered by all reference nodes, where the smallest estimation error is 0.15 m. As mentioned earlier, the signal-to-noise ratios of the multiple measurements of RSSI patterns can be increased if the antenna of the reference node rotates one more complete cycle. As shown in Figure 7(b), the estimation results yielded by the proposed algorithm are more accurate. The averaged bias is 1.30 m, the standard deviation of the bias is 0.66 m, the maximal bias is 2.93 m, and the minimal bias is 0.07 m.

To further compare the performance of the proposed method with other quantitative techniques, multidimensional scaling (MDS), maximum-likelihood estimator (MLE), and hybrid of MDS and MLE (MDS-MLE) were applied to the same deployment structure. The results yielded by the proposed algorithm and different weighting methods in [20], [40], [41], and [42] are summarized in Table 2. We can see that in the MDS and MLE solutions, the bias effect is still very significant. The two-stage MDS-MLE methods greatly alleviate the bias effect, but the biases are still around 5 m. The proposed algorithm outperforms these methods with significantly smaller bias.

**Figure 8.**
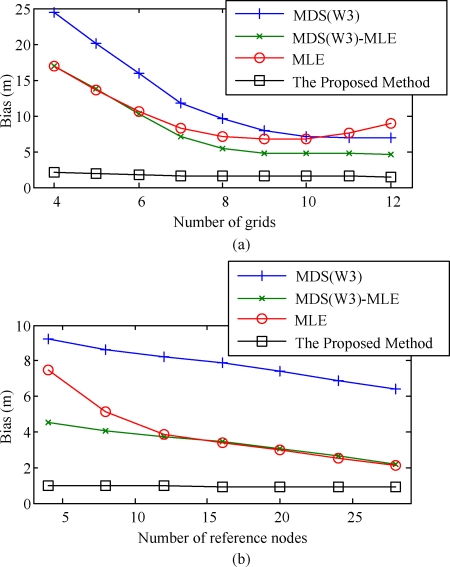
Bias performance of the proposed algorithm and previously proposed methods (a) versus the number of grids and (b) versus the number of reference nodes.

From the simulation results shown in Figure 8(a), we can see that MDS, MLE, and MDS-MLE have better performances when the number of grids increases. The MDS-MLE method is able to consistently improve the results yielded by MDS after removing the modeling error of MDS. Different from these methods, the proposed algorithm yielded smaller estimation bias when the number of sensor nodes becomes large. However, the results yielded by the proposed algorithm are significantly more consistent than those in the previous methods. The same trend also appears in the simulation results shown in Figure 8(b), where all reference nodes are uniformly deployed at the grid points on the border of the network. This would be a good feature of the proposed algorithm since it shows the stability of the proposed algorithm.

### 5.2. Performance Evaluations in Real-World Scenarios

**Figure 9.**
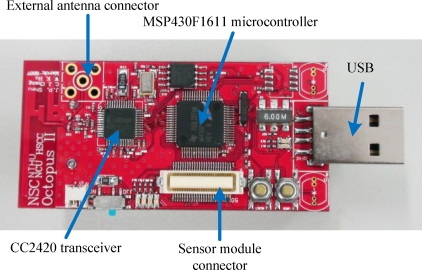
Octopus II-A sensor node utilized in this study.

In this subsection, we apply the proposed algorithm to real-world scenarios using a WSN platform. The sensor nodes used in this study are Octopus II-A [43], as shown in Figure 9. Octopus is an open-source visualization and control tool for sensor networks developed in the TinyOS 1.x environment [44]. It consists of a MSP430F1611 microcontroller, a USB interface, and an onboard inverted F and SMA type antenna. Its specification is very similar to the Tmote-Sky sensor node [45]. CC2420 is an RF transceiver responsible for measurements of the RSSI patterns.

**Figure 10.**
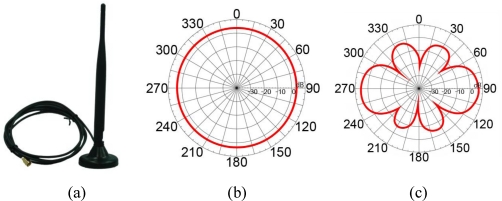
Specification of the omnidirectional antenna utilized in this study. (a) Maxim AN-05DW-S Antenna [46] that is connected to all sensor nodes used in this study, and the radiation patterns of the antenna in the (b) H-plane and (c) E-plane.

In order to simplify the problem, we connected an external antenna to each sensor node. The antenna is an omnidirectional 5 dBi high gain antenna (Maxim AN-05DW-S [46]) as shown in Figure 10(a).

**Figure 11.**
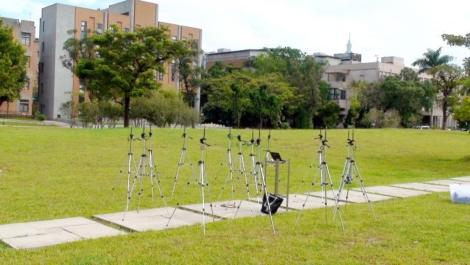
Testing environment of the experiment located on the campus of the National Taiwan University.

It is designed to support 2.4 GHz RF signals and the most popular protocols defined by IEEE 802.11b and 802.11g. The radiation patterns of the antenna in the H-plane and E-plane are depicted in Figures 10(b) and 10(c) [46], respectively. The onboard antenna of the sensor nodes is disabled in these field experiments. All sensor nodes are coupled with an external antenna, and the antenna is set at an upward oriented position. The reference node is coupled with a same type of antenna using the configuration shown in Figure 2(b). The testing environment, which is shown in Figure 11, is located on the campus of the National Taiwan University.

In real-world scenarios, it is impossible to construct a reference standard pattern for a reference node under all of the possible distances and orientations of the external antenna. We measured the values of RSSI when a sensor node is moved away from the reference node by five individual distances (1.8 m, 5 m, 10 m, 13 m, and 18 m).

**Figure 12.**
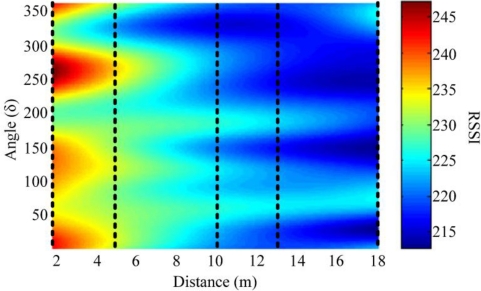
Reference standard RSSI pattern measured from the experiment in a real-world scenario, where the dash lines are obtained by 1,000 repeated experiments.

In order to save electric energy of all sensor nodes, we measured the RSSIs when the azimuths of the external antennas of the reference node is 0°, 30°, 60°, …, and 330°. The cubic spline interpolation technique is used to predict the RSSI values at unmeasured azimuths. Base on these results, we used a 2nd order polynomial curve fitting model to identify the RSSI values at unmeasured distances. The constructed RSSI pattern is depicted in Figure 12.

**Figure 13.**
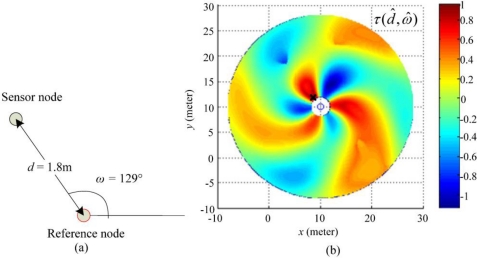
Experimental result for single reference node scenario. (a) Deployment arrangement of the sensor node and reference node in the scenario for single reference node. (b) Estimation result using the proposed robust correlation. The estimated coordinate of the sensor node is annotated by black cross (×).

First, we used the proposed algorithm to localize a sensor node in a single reference node scenario. The deployment arrangement is depicted in Figure 13(a), where the coordinate of the reference node is (10, 10), the sensor node and the reference node are separated by 1.8 m, and the azimuth of *s* to *r* is 129°. The robust correlation *τ*(*d̂*, *ω̂*) estimated from the measured RSSI pattern is shown in Figure 13(b). In this test case, the maximum correlation is presented at *τ*(1.9 m, 128°), which implies that the estimated coordinate of the sensor node is (–1.1957, 1.4766). By comparing the estimation result with the true position of the sensor node, the estimation bias is 0.1051 m.

**Figure 14.**
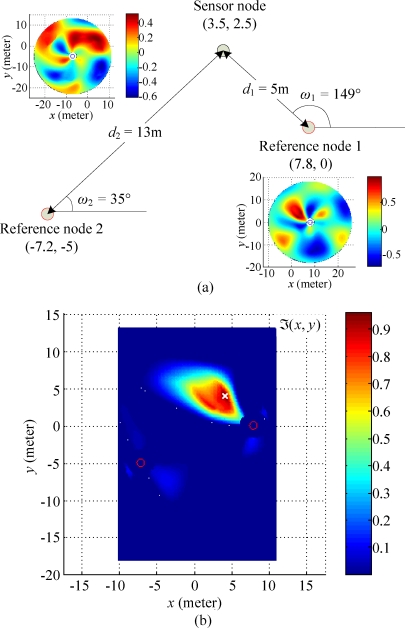
Experimental result for two reference nodes scenario. (a) Deployment arrangements of the sensor node and the reference node in a scenario for two-reference nodes. (b) Overall solution space with coordinates of reference nodes (red circles 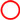) and estimated coordinate (white cross ×) of the sensor node.

In the two-reference nodes scenario, two reference nodes are deployed at individual coordinates (7.8, 0) and (−7.2, −5). The true position of the sensor node is at (3.5, 2.5). The deployment arrangement is depicted in Figure 14(a). By using the collaborative localization scheme previously introduced in Section 4, an overall solution space ℑ(*x, y*) can be constructed as shown in Figure 14(b). The centroid coordinate of ℑ(*x, y*), which can be used to indicate the potential location of the sensor node, is located at (3.8, 3.6). By comparing the estimation result with the true position of the sensor node, the estimation bias is 1.14 m. The estimation bias in the two-reference nodes scenario is larger than that in single reference node scenario because the distances between sensor node and reference nodes in the previous scenario are significantly larger than that in the latter one.

In addition, due to unknown environment conditions (e.g., standing electromagnetic waves, and electromagnetic absorption or interference), the reference standard RSSI pattern, as shown in Figure 12, was not changed much when the sensor node and the reference node are separated by around 13 m. Therefore, noise may influence the localization accuracy of the proposed method when the sensor node and the reference node are separated by around 13 m. Therefore, in the two-reference nodes scenario, the localization accuracy of the proposed method was decreased since the reference node 2 and the sensor node were distanced by 13 m. Such bias can be significantly reduced by increasing the number of antenna rotations or adding another reference node to assist the localization process.

**Figure 15.**
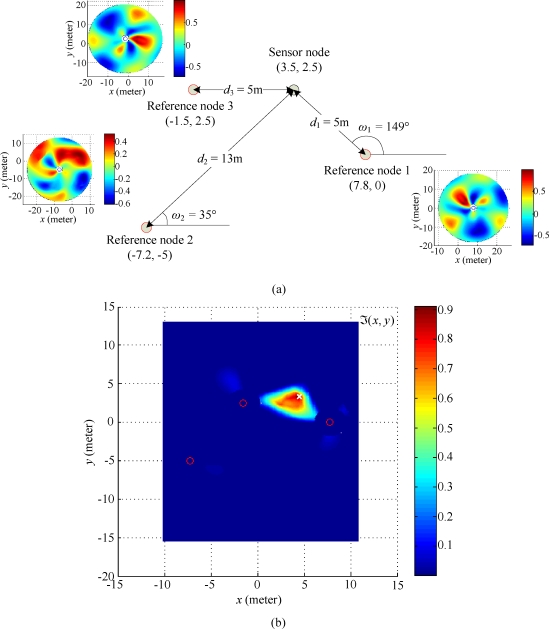
Experimental result for three reference nodes scenario. (a) Deployment arrangement of the sensor node and reference node in the scenario for three-reference nodes. (b) Overall solution space with coordinates of reference nodes (red circles ○) and estimated coordinate (white cross ×) of the sensor node.

If we want to improve the localization accuracy obtained in two-reference nodes scenario, another reference node may be added at the coordinate (–1.5, 2.5). This leads a three-reference nodes scenario, as shown in Figure 15(a). The robust correlations *τ*(*d̂*, *ω̂*) estimated from individual reference nodes are merged into an overall solution space ℑ(*x*, *y*) as illustrated in Figure 15(b). The estimation result shows that the coordinate of the sensor node is (3.3, 2.5). Comparing the estimation results with the true position of the sensor node, the estimation bias when using the three-reference nodes scenario is significantly reduced to 0.2 m. These findings suggest that more reference nodes deployed in the network can improve the estimation accuracy when the proposed localization algorithm is employed.

**Figure 16.**
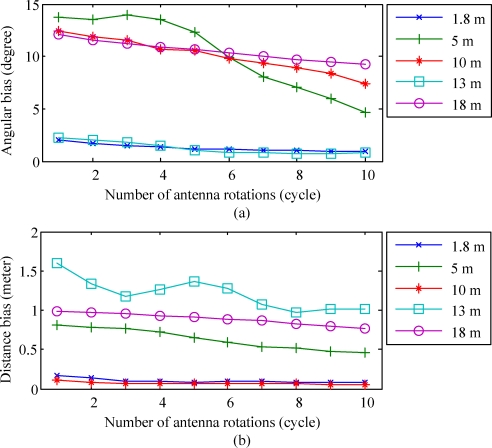
(a) Average angular biases and (b) averaged distance errors yielded by the proposed algorithm versus different number of antenna rotations (cycle).

The association between the angular bias and the number of antenna rotation has been also examined. We have conducted the same experiments 50 times to estimate the average angular bias under different number of antenna rotations. The experimental results are depicted in Figure 16(a). It can be seen that the accuracy of the angular estimation is improved as the number of antenna rotations increases. This implies that if multiple measurements of the RSSI pattern are available, the performance of the proposed algorithm can be enhanced. We also analyze the relation between the estimation bias and the number of antenna rotations. A total of 50 repeated experiments have been conducted to estimate the averaged distance error resulted from the proposed algorithm. The results are illustrated in Figure 16(b). It is apparent that if the number of antenna rotations is increased, the distance error yielded by the proposed algorithm will be reduced.

## 6. Conclusions

An RSSI-based collaborative localization method that makes use of the irregularity of the EM wave is proposed. First, we coupled external low-cost omnidirectional antennas with sensor nodes and reference nodes using specific antenna configurations. The antenna of the reference node rotates in the horizontal plane to measure the RSSI pattern between the sensor node and the reference node. A robust estimation technique is also presented to analyze the RSSI patterns obtained by the reference node. The RSSI pattern might involve some noise caused either by antenna specification or by environmental conditions. By using the proposed antenna configuration to generate multiple RSSI measurements, the signal-to-noise ratio of the RSSI pattern can be increased. The proposed algorithm is thus able to provide the localization results with higher precision. In addition, a collaborative localization scheme is presented to integrate the information obtained by multiple reference nodes.

The proposed algorithm has been evaluated through computer simulations and real-world experiments. Several algorithms (including MDS, MLE, and MDS-MLE) that use different weighting schemes are also applied to the same simulation cases. The simulation results show that the proposed algorithm outperforms these algorithms with estimation bias smaller than 1 m. The proposed algorithm is also examined in real-world scenarios using different number of reference nodes. The estimation bias is around 0.1 m, 1.14 m, and 0.2 m, respectively. Averaged estimation biases are also analyzed and reported.

Both computer simulations and real-world experiments have confirmed that the proposed algorithm is not perfect but it is a significantly advanced method than other ones. The proposed algorithm uses low-cost omnidirectional antennas to achieve accurate localization, and it does not require special information that can only be measured by special instruments (e.g., ultrasound devices, directional antennas) in order to localize a sensor node in the network. Finally, how to determine the speeds and 3-D locations of the moving sensor nodes and how to perform localization in the presence of security threats in WSNs, are left as our future works.

## Figures and Tables

**Table 1.** Simulation parameters.

**Simulation Parameters**	**Parameter Value**
Size of sensor field	80 m × 80 m
Number of grids	8
Number of reference nodes	4
Path-loss exponent *α*	3
Standard deviation of noise in Ω_<_*_r_*_,_*_s_*_>_(*δ*)	6 dB
First meter (*d*_0_ = 1) RSS *P*_0_	−30 dBm
RSS detection threshold	−80 dBm
Neighborhood selection threshold	−75 dBm

**Table 2.** Performance statistics of the proposed algorithm and different methods using previous proposed weighting schemes^*^ W1 [20], W2 [40], W3 [41] and W4 [42].

**Method**	**Bias (m)**	**STD (m)**	**RMSE (m)**
MDS(W1)	8.40	15.26	17.41
MDS(W2)	12.23	10.96	16.42
MDS(W3)	9.18	10.97	14.30
MDS(W4)	9.03	12.8	15.67
MLE	6.81	13.56	15.18
MDS(W1)-MLE	5.93	12.39	13.73
MDS(W2)-MLE	5.44	9.06	10.57
MDS(W3)-MLE	4.68	8.89	10.05
MDS(W4)-MLE	5.19	9.96	11.24
Proposed Method (1 cycle)	1.89	1.31	3.75
Proposed Method (2 cycle)	1.30	0.66	2.43

* Results of the previous studies were reported in [22].
